# Investigating Neural Sensorimotor Mechanisms Underlying Flight Expertise in Pilots: Preliminary Data From an EEG Study

**DOI:** 10.3389/fnhum.2018.00489

**Published:** 2018-12-13

**Authors:** Mariateresa Sestito, Assaf Harel, Jeff Nador, John Flach

**Affiliations:** Department of Psychology, Wright State University, Dayton, OH, United States

**Keywords:** affordances, embodied cognition, EEG, flight expertise, mirror neuron system, mu rhythm suppression, perception

## Abstract

Over the last decade, the efforts toward unraveling the complex interplay between the brain, body, and environment have set a promising line of research that utilizes neuroscience to study human performance in natural work contexts such as aviation. Thus, a relatively new discipline called neuroergonomics is holding the promise of studying the neural mechanisms underlying human performance in pursuit of both theoretical and practical insights. In this work, we utilized a neuroergonomic approach by combining insights from ecological psychology and embodied cognition to study flight expertise. Specifically, we focused on the Mirror Neuron system as a key correlate for understanding the interaction between an individual and the environment, suggesting that it can be used to index changes in the coupling of perception-action associated with skill development. In this study, we measured the EEG mu suppression as a proxy of the Mirror Neuron system in experts (pilots) and novices while performing a distance estimation task in a landing scenario. To survey the specificity of this measure, we considered central, parietal and occipital electrode pools and analyzed alpha (8–13 Hz) and beta (18–25 Hz) rhythm bands. We hypothesized that in experts vs. novices, specific neural sensorimotor brain activity would underpin the connection between perception and action in an in-flight context. Preliminary results indicate that alpha and beta rhythm suppression was area-specific irrespective of groups, present in the central electrodes placed over the motor areas. Group analysis revealed that specifically alpha mu rhythm, but not beta, was significantly more suppressed in pilots vs. novices. Complementing these findings we found a trend in which the strength of mu suppression increased with the sense of presence experienced by the pilots. Such sensorimotor activation is in line with the idea that for a pilot, a distance judgment is intimately associated with the function of landing. This reflects the ability to use optical invariants to see the world in terms of the capabilities of the aircraft (e.g., reachability and glide angle). These preliminary findings support the role of embodied simulation mechanisms in visual perception and add important insights into a practical understanding of flight expertise, suggesting sensorimotor mechanisms as potential neuro-markers.

## Introduction

“*Assume you are at 2,000 feet, somewhere over the country, looking at a field in which you intend to land. Can you reach it? You measure (mentally) its angular distance from the horizon, and you judge that it lies about 15 degrees below the horizon. You know that in your ship you can reach any spot that lies 10 degrees below the horizon, or steeper. Hence you know that you can comfortably reach that field*.”

(*Stick and Rudder*, Langewiesche, 1944, p. 272).

Researchers have recognized that an intimate coupling of perception and action is essential to pursuing intentions and satisfying needs (Paillard, 1991; Previc, 1998). Imagine it is a hot summer day, and you have spent the whole day hiking in the hilly countryside of Tuscany, Italy. You run out of water supply, and at a certain point you come across a creek: such a find will likely invite you to stop to relieve your thirst. Normally this circumstance would not evoke such an action; however, the need to rehydrate may induce you to promptly notice this possibility in the environment around you. In the same vein, it is fairly easy to observe how an “invitation to action” can be found in the environment itself. This can be found in several ordinary architectural circumstances: stairs invite walking on them, a cup invites being grasped, and every time we look around, we are somehow conscious of what is reachable and what is not (Gallese and Gattara, 2015).

Likewise, in aviation visual cues present in a scenario may be utilized to guide action. This becomes clearer if you consider the task of safely landing an airplane. As described by Langewiesche, when a pilot is being asked to define the position of an object or an airfield, this will typically result in a judgment that takes into account the angle-under-the-horizon as a unit of measure. Indeed, as Langewiesche insightfully argues:

*“The experienced pilot sees the ground much in the same manner in which the astronomer sees the heavens – in terms of “angular distances” rather than height, depth, and distance in the usual sense. Here is what this means. You ask somebody, “I can’t see that star you’re talking about; where is it? (…) or that “bird”, or “that airplane”; in short, you ask him the location of a point on the heavens. He will not be able to answer concisely. He will hem and haw and say “there” and point (…). Not so the astronomer or the artillery man or the mariner. He will say effortlessly and precisely, “The airplane is 45 degrees above the horizon (…).” He sees the heavens as a huge hemispherical bowl over him, on the inner surface of which the stars, clouds, birds, and airplanes are painted. It is obviously only a fiction, but it works; it allows him to measure and describe the apparent position of any point on the heavens with great accuracy* (Langewiesche, 1944, pp. 269–270).

**FIGURE 1:**
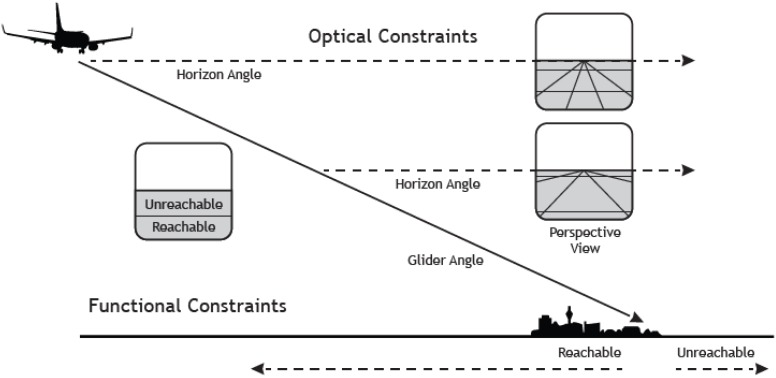
The figure depicts a scenario where an airplane is landing at an airport. As the aircraft descends along a fixed approach path the horizon will always be at the pilot’s eye-height (see the dotted straight line) and the focus of expansion (indicating the point on the ground toward which the aircraft is moving) will be fixed. Thus, in a stabilized approach the relation between horizon and aim point is invariant. Knowing the safe or maximal glide angle as a functional property of a particular aircraft allows skilled pilots to directly see “reachability” as a function of angular position relative to the horizon. Positions below the safe glide angle are “reachable,” and positions above the glide angle (nearer the horizon) are beyond the glide capabilities of the aircraft. Reproduced with permission from Flach and Voorhorst (2016).

But what is the critical property of this measure? Critically, in a landing task, the angle-under-the-horizon has the functional property to express the location of an aircraft in terms of glide angle to a specific point on the ground (Figure 1). This importantly, allows the pilot to directly differentiate between locations on the ground that are within the glide range and that can hence be reached with the airplane, from those that are outside the glide range, that are hence unreachable (Flach and Voorhorst, 2016; Figure 1).

It follows that for a pilot, a seemingly perceptual task such as distance judgment is framed in terms of the action capabilities of an aircraft (e.g., the glide angle). Thus, for skilled pilots perception is tightly linked to both the action constraints of the vehicles in relation to safe locomotion to make a soft contact with the ground. This example highlights how visual information can be processed in relation to the possibilities for action (i.e., affordances, Gibson, 1979). However, in the case of aviation, the action capabilities of the aircraft would not be generally experienced by most humans. It is hypothesized that these affordances would only be seen by pilots, who had discovered the action constraints through extensive flying experience.

Over the last decade, the efforts toward unraveling the complex interplay between the brain, body, and environment have set a promising line of research that utilizes neuroscience to study human performance in natural work contexts such as aviation. Thus, a relatively new discipline called neuroergonomics is holding the promise of studying the neural mechanisms underlying human performance in pursuit of both theoretical and practical insights (Parasuraman and Rizzo (eds).,  2008). In this work, we utilized a neuroergonomic approach by combining insights from ecological psychology and embodied cognition to study flight expertise. Specifically, we focused on the Mirror Neuron system as a key neurophysiological correlate for understanding the interaction between an individual and the environment, arguing that it can be utilized to capture changes in the coupling of perception-action associated with skill development.

Mirror Neurons have the peculiarity to discharge both when a certain action is executed, and when a similar action is observed (Gallese et al., 1996; Rizzolatti et al., 1996). Such evidence suggests the existence of neurons that dually reflect joint properties of the perceptual motor coupling, thus providing a neural substrate that directly couples action and observation. Mirror Neuron activity hence reflects the tight coupling between perception and action which revitalizes the Gibsonian ecological theory of direct specification of affordances by optical invariants (Gibson, 1979).

From this framework, it can be easily argued that vision is in fact a multimodal endeavor: the involvement of actual motor capabilities underpinning perceptual dimensions has been indeed found in previous studies for actions, emotions, and corporeal sensations, as well as perception of art and architecture (Freedberg and Gallese, 2007; Jelic et al., 2016). In this respect, several studies demonstrate how experience and skill involved with particular actions affect the use of motor simulation to process observed action in different domains (see Calvo-Merino et al., 2004, 2006; Aglioti et al., 2008 for evidence reported in expert dancers and basketball players; Haueisen and Knösche, 2001; Haslinger et al., 2005; Landau and D’Esposito, 2006 for findings reported in pianists). This process takes place because experts like dancers and musicians hold the experience related to particular dance moves or finger movements in the form of actual encoded motor chains. These skilled motor repertoires can be indeed evoked through observation – hence the experts can “resonate” with that observed action they are skilled at. Thus, it appears that previous experience will modulate perception-action mechanisms at a brain level, and that this brain activity might be used to distinguish experts from non-experts (Yang, 2015). In addition, recent neuroscientific findings highlighted the role of sensorimotor areas in the appreciation of works of art (Umilta’ et al., 2012; Sbriscia-Fioretti et al., 2013), demonstrating that motor system activation through embodied mechanisms occurs even for implied actions like cuts on a canvas and brush strokes. An interesting aspect of these studies is that they utilized neurophysiological responses that included mu rhythm suppression. Mu is constituted by a range of electroencephalography (EEG) oscillations generally recorded at a frequency of 8–13 Hz from scalp electrodes corresponding to the sensorimotor brain areas (Hobson and Bishop, 2016). Normally, when an individual is at rest the neurons in the sensorimotor cortex fire in synchrony. Conversely when an individual executes, observes, or imagines herself performing an action, the firing of these neurons become desynchronized. This desynchronization leads to mu power reduction (Pfurtscheller et al., 1997). Because mu suppression occurs both when a person performs and observes an action, this physiological measure has been considered as a legitimate indicator of the Mirror Neuron system activity in humans (Muthukumaraswamy and Johnson, 2004; Muthukumaraswamy et al., 2004; Pineda, 2005; Oberman et al., 2007; Perry and Bentin, 2009; Hobson and Bishop, 2017).

Interestingly, motor resonance has been also demonstrated in the domain of skilled performance related to flight expertise (Callan et al., 2012, 2013). When a pilot observes someone else flying from a third person perspective, this scenario evokes in his brain those motor actions performed on the flight controls that are necessary in order to accomplish those maneuvers. In line with Gibson’s notion of affordances (1997), such a motor resonance allows the expert to intuitively (i.e., pre-reflectively) understand what is going on in such a scenario.

The aim of the present study was to investigate the extent to which neural sensorimotor activity (i.e., mirror neuron activity; measured by means of mu rhythm suppression) is detectable in experienced participants (i.e., pilots) vs. inexperienced participants (i.e., novices) during visual perception of simple static images depicting a landing scenario. In this context, neural sensorimotor activity is hypothesized to reflect the discussed connection between perception and action in an in-flight situation that normally occurs in experts: as Langewiesche (1944) indeed insightfully emphasized in the quotes reported, when a pilot is asked to gauge the distance of the runway in a landing scenario, he will automatically frame this task in terms of the action constraints associated with a landing task, while this will not occur in non-experts. Using EEG, we measured the intensity of mu rhythm suppression from the scalp central electrode sites during the observation of artificially created landing scenarios while performing a distance estimation task. Notably, the use of a simple distance estimation task enabled us to easily include in the study a non-expert population to be compared with pilots, avoiding the potential complications that may arise from including technical flight-related aspects in the task.

Considering that both mu and alpha are measured in the same frequency band (8–13 Hz), one of the main concerns in studies utilizing mu suppression is whether it can be reliably separated from changes in alpha activity coming from other brain regions. It has been indeed reported in previous studies how generic alpha activity is blocked or attenuated by attention and more generally by mental effort (Niedermeyer and da Silva, 2005); indeed, more task difficulty elicits more alpha suppression (Gevins et al., 1997; Stipacek et al., 2003). What makes alpha and mu distinguishable is their different topography and reactivity. While alpha arises in the posterior and occipital brain areas, mu arises specifically from the sensorimotor area (Gastaut, 1952). Accordingly, while modulations in the mu power are typically ascribed to activity in the sensorimotor cortex, alpha power is thought to index attentional engagement (Pfurtscheller, 1992; Klimesch, 1999; Cochin et al., 1999). Because of this potential confound, in the current study we controlled for the posterior alpha activity by contrasting occipital as well as parietal electrode sites (Perry and Bentin, 2009; Frenkel-Toledo et al., 2013; Hobson and Bishop, 2016).

Another aspect to consider pertains to the fundamental multispectral nature of mu rhythm (Tiihonen et al., 1989; Cochin et al., 1999; Babiloni et al., 2002; Nam et al., 2011, see Pineda, 2005). It has been indeed reported in previous studies (Hari, 2006; Avanzini et al., 2012) how the rolandic mu rhythm consists of two main frequency components: one around 10 Hz (alpha), and the other around 20 Hz (beta). It has been suggested that mu recorded in the alpha band is more related to sensory processing rather than motor activity, while changes in beta power, but not mu, are indicative of motor cortex activity (Pfurtscheller et al., 1994, 1996; McFarland et al., 2000; Orgs et al., 2008; Ritter et al., 2009; Coll et al., 2015). Supporting this claim, Avanzini et al. (2012) showed how the velocity profile of observed movements strongly correlates with beta power, but not alpha. Also, other recent studies reported how the velocity of executed as well as imagined movements are able to modulate the beta frequency band (Kilner et al., 2003; Press et al., 2011). Given the need for further investigation of beta-band responses (Fox et al., 2016), to probe the specificity of mu response we opted to investigate the reactivity of both sensorimotor frequencies bands: alpha (8–13 Hz) and beta (18–25 Hz).

In addition, subjective ratings on closeness with a realistic scenario, degree of familiarity, sense of presence and amount of movement were collected in order to explore correlations with these explicit measures with implicit brain activity. Because we did not use virtual reality in our study, we adopted the sense of presence rating as it has been considered as an index of a physiological state which reproduces realistic behaviors and physiological responses as if the participant was experiencing a real-life situation (Vecchiato et al., 2015).

Based on prior studies, we hypothesized that in pilots previous experience with a landing scenario due to flight expertise will modulate perception-action mechanisms at a brain level, leading to increased mu-suppression relative to novices, and that this increased activity would be correlated with other subjective measures quantifying participants’ immersion in the task.

## Materials and Methods

### Participants

Nineteen volunteers (11 pilots, hereafter defined Airmen Group, AG; 8 novices, hereafter defined Novice Group, NG) were recruited in the local community and in the flying clubs present in the Dayton, OH, United States area. Two participants (*N* = 2, AG) were excluded because of excessive muscular activity during the experimental session.

Eligible volunteers were assessed in their interest in participating to the study as well as in their ability to give a valid consent to entering the study. Subsequently, all consented participants underwent a first screening to determine their eligibility for the study and only those meeting the inclusion criteria and not having exclusion criteria were included. Volunteers included in the NG were individuals with no flight expertise as well as with no experience as a passenger in a small airplane. Participants included in the AG were recreational and professional pilots with different levels of expertise. No participants reported the presence of any current and/or past neurological or psychiatric disorder and drugs/alcohol abuse, and all had a normal or corrected to normal vision.

General information aimed at controlling for potential variables impacting performance and brain measures were acquired from participants and included gender, age, handedness and education. For measuring pilot’s expertise, the number of flight hours [under Visual Flight Rules (VFR), and Instrument Flight Rules (IFR)] were recorded. In addition, information about flight experience as a passenger and video game experience were collected, as they imply possible exposure to flight scenarios and therefore may potentially acts as confounds in the experiment. Demographic characteristics of the samples considered in the final EEG data analysis are the following: AG: mean age 44.89, DS ± 15.61; mean education 17.11, DS ± 1.90; 9 males; 1 left-handed; NG: mean age 30.63 DS ± 13.09; mean education 17.38, DS ± 1.30; 7 males; 1 left-handed. Reported mean AG total flight time is 1,547 flight hours (DS ± 2,319.86), of which VFR: mean 1,244.44; DS ± 1,589.22; IFR: mean 302.56; DS ± 787.76. AG and NG did not differ with respect to age, years of education, and video games experience (all *p*_s_ > 0.05). Considering that studying laterality effects was not among the aims of this study, we included both right- and left-handed participants. Handedness variable in any case, was well balanced in the two groups (1 left-handed for AG, 1 left-handed for NG).

Written informed consent was obtained from all participants before entering the study. The institutional review board of Wright State University approved the study protocol, which was carried out according to the ethical standards of the 2013 Declaration of Helsinki.

### Stimuli

**FIGURE 2:**
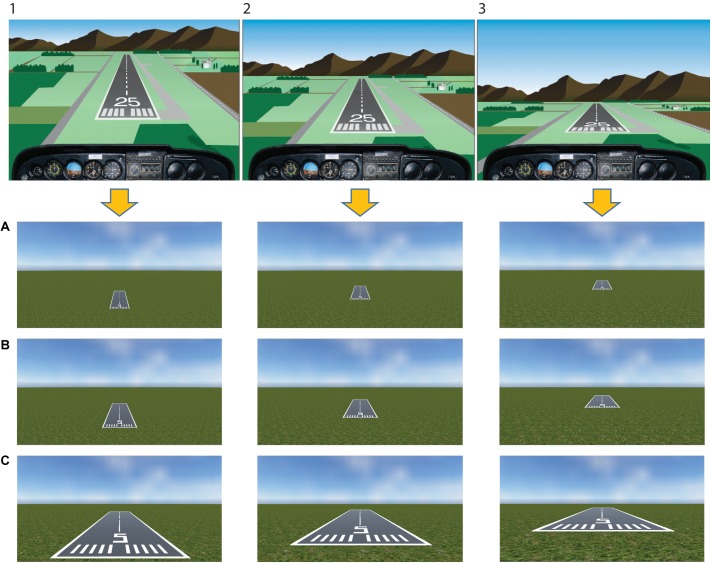
Stimuli. Stimuli have been built considering three different glide paths as seen from the cockpit (upper panel:1, too high; 2, proper descent angle; 3, too low), that correspond to three different height/distance ratios reported in the lower panel (0.4, high glide path; 0.3, on the glide path; 0.2, low glide path, respectively). In the lower panel, three different distances from the runway were included (**A**, 150 m/689 ft; **B**, 90 m/295 ft; **C**, 30 m/98 ft). The upper panel figure has been adapted with permission from U.S. FAA-H-8083-3B 2016.

Twenty-seven static images depicting a typical landing scenario were constructed using the Unity game engine (63° field of view)^1^. Stimuli represented three runways being seen from three increasing distances (30 m/98 ft, 90 m/295 ft, 150 m/689 ft) and 3 height/distance ratios corresponding to three different glide paths (0.2 = low glide path, 0.3 = on the glide path, 0.4 = high glide path) (see Figure 2 for stimuli example). Based on this, in a typical low glide path view (i.e., 0.2 height/distance ratio), the approach is too low and the runway cannot be reached in a powerless glide. A typical correct landing approach is represented in a scenario adopting the 0.3 height/distance ratio (i.e., on the glide path). The other possible scenario is represented by a high approach (i.e., 0.4 height/distance ratio), where the landing spot is shifted forward. Stimuli and related reachability of the runway in a powerless glide were independently rated in a previous validation study by a different sample of pilots (see the Supplementary Data Sheet 1).

### Experimental Procedure

Participants were comfortably seated facing a 27-inch computer monitor located at a distance of 65 cm in a dimly illuminated room-sized electromagnetically shielded booth. Participants were told to find a comfortable position, minimize their movements during the experiment and to stay as relaxed as possible. During the experiment participants observed the presented landing scenes and were asked to perform two distinct tasks (counterbalanced across block sessions): a Distance Task (DT, i.e., experimental task) and an Identification Task (IT, i.e., control task). In the DT, participants were asked to gauge the distance of the runway number. In the IT, they were asked to identify the runway number.

**FIGURE 3:**
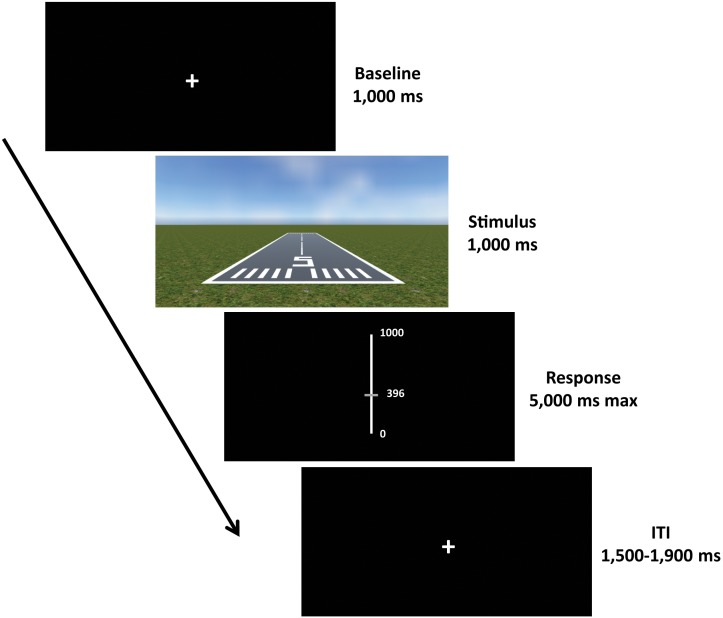
Experimental paradigm. Each trial started with a fixation cross (baseline) with a duration of 1000 ms, followed by the presentation of the scene for 1000 ms. Afterward, a visual analog scale (VAS) was presented for 5000 ms (maximum duration), where participants were required to give their response. The response was provided by moving the slider up and down using the computer mouse and then confirming the choice by clicking the left button. Subsequently, an inter-trial interval (ITI) with a varying duration of 1500–1900 ms was presented before the next trial begins.

Each trial started with a fixation cross (baseline) presented for a duration of 1000 ms, followed by the presentation of the scene for 1000 ms. Afterward, a visual analog scale (VAS) appeared for 5000 ms (maximum duration), during which participants were required to provide their response using the computer mouse. Subsequently, an inter-trial interval (ITI) with a varying duration of 1500–1900 ms was presented, in order to allow brain activity related to motor response to subside before the next trial begins (Cohen, 2014; Wamain et al., 2016; Figure 3).

Twenty-seven stimuli were randomly presented, repeated 10 times each for a total amount of 270 stimuli for each task (DT, IT).

### Behavioral Rating

At the end of the EEG recording session, participants were asked to rate the scenes presented in the experiment for their (1) Familiarity (“How familiar are you with the scenes presented?,” scored from 0 to +9); (2) closeness with a realistic scenario (“How realistic did you perceive the scenes?,” scored from 0 to +9); (3) Presence (“To what extent did you have the sense of being in the scene? That is, to what extent were there times during the experience when the scene became reality for you and you almost forgot about the real world in which the whole experience was really taking place?” (see Vecchiato et al., 2015), scored from 0 to +9); (4) Amount of movement (“How much movement did you perceive in the scenes?” (see Umilta’ et al., 2012), scored from 0 to +9). The sense of presence is defined as the illusory sensation of being physically in the scene, usually utilized in the experience of virtual reality scenarios (Diemer et al., 2015). The amount of movement is intended to provide an explicit measure of possible action-related visual cues that may be perceived in the scenarios.

### EEG Recording

Electroencephalography data were recorded using a BioSemi ActiveTwo system (BioSemi B.V., Amsterdam, Netherlands) with 64 Ag/AgCl electrodes and a sampling rate of 1024 Hz. A BioSemi headcap (BioSemi B.V., Amsterdam, Netherlands) was used to position the 64 EEG electrodes on the scalp, according to the international 10–20 system. For EEG reference, two external electrodes were placed on the right and left mastoid bones. In addition, four external electrodes were used to measure vertical and horizontal electrooculography (EOG). Electrode impedances were kept under 5 kΩ.

A standard computer mouse and Presentation software (NBS)^2^ were used to control stimuli delivery, and all event markers were sent to Brain Vision Recorder Software (Brain Products)^3^. Participants’ motion was monitored by a video camera.

### Behavioral Data Analysis

The rating score of each participant was first averaged on the basis of distance and height/distance ratios. The corresponding averaged rating scores were then collapsed based on group, and group differences were tested using a *t*-test (two tailed, *p* < 0.05).

### EEG Data Processing and Analysis

Raw EEG data were subjected to 1–30 Hz band-pass filter, and then re-referenced to the average of the two mastoids. ICA-based artifact correction procedure (Jung et al., 2000; but see also Delorme and Makeig, 2004) as implemented in Brain Vision Analyzer software (Brain Products)^4^ was used in order to correct eye movements. Remaining artifacts exceeding ± 100 μV in amplitude were rejected. In order to reduce the possibility of attentional effects due to initiation and termination of the block session, the first and the last stimulus of each block were discarded from the analysis (see Pineda and Oberman, 2006 and Perry and Bentin, 2009 for a similar practice).

Following this procedure, 88.60% of trials of the DT condition (NG: 89.65%; AG: 87.54%) and 88.49% of trials of the IT (NG: 88.48%; AG: 88.50%) were retained for the subsequent analyses.

Electroencephalography was segmented into condition segments and relative baselines. For each 1 s epoch, the integrated power recorded in the 8–13 Hz range (alpha rhythm range) and 18–25 Hz range (beta rhythm range) was calculated by means of a Fast Fourier Transform (FFT) performed at 0.5 Hz intervals (based on 2048 data points per segment, utilizing a Hanning window).

In this way, the dependent variable considered in the EEG statistical analyses was the ratio of the power during the task condition (i.e., DT activity minus control IT activity) relative to the power during the baseline condition. We chose to adopt a ratio in order to control for variability in absolute mu power as a result of individual differences like electrode impedance and scalp thickness (Pineda and Oberman, 2006). Moreover, as ratio data are usually non-normal, a log transform was performed. A log ratio of less than zero means suppression, while a value of zero means no suppression and values greater than zero indicate enhancement.

**FIGURE 4:**
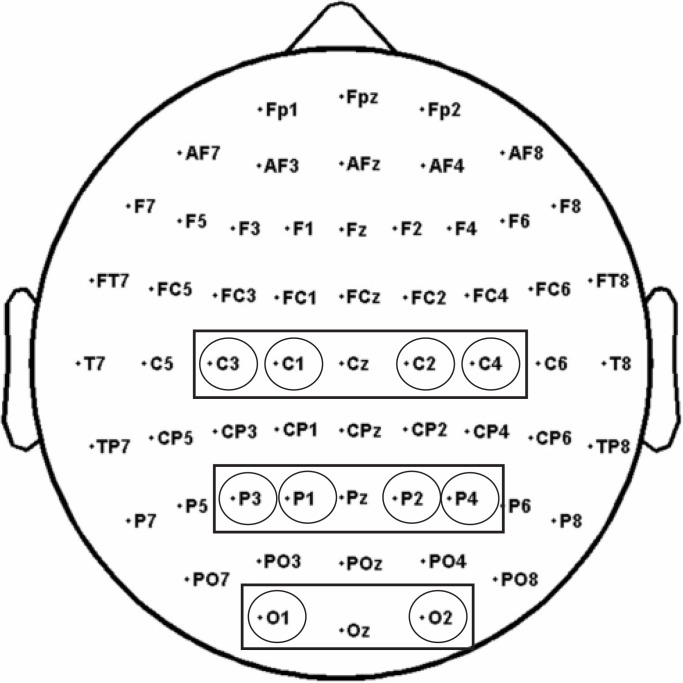
The analysis of alpha and beta rhythms was performed on three electrode pools: a Central pool (comprising C3, C1, C2, C4), a Parietal pool (comprising P3, P1, P2, P4), and an Occipital pool (O1, O2).

Based on previous studies on mu suppression distribution and Mirror Neuron activity (Perry and Bentin, 2009; Avanzini et al., 2012; Umilta’ et al., 2012; Frenkel-Toledo et al., 2013), three clusters of electrodes were selected and considered for the analyses: a Central cluster comprising C3, C1, C2, C4, a Parietal cluster comprising P3, P1, P2, P4, and an Occipital cluster comprising O1, O2 to control for the posterior visual alpha (Hobson and Bishop, 2017; Figure 4).

First, in order to assess the topography of alpha and beta rhythms irrespective of groups during the presentation of the stimuli, a three-way (Electrode Pool: Central, Parietal, Occipital) repeated measures ANOVA was performed separately for alpha and beta rhythm activity, with Electrode Pool as within-participants factor. Afterward, data were collapsed based on group and group differences were investigated using a *t*-test for the electrode pools that turn out to be significant (two tailed, *p* < 0.05).

For all performed analyses, *p* values < 0.05 were considered to be statistically significant. As index of effect size we reported eta squared values (η^2^_p_) (Cohen, 1988). *Post-hoc* comparisons (Bonferroni corrected for multiple comparisons) were applied on significant main effects and interactions for ANOVAs.

## Results

First, we verified that our variables were normally distributed by means of visual inspection of histograms and the application of the Kolmogorov–Smirnov test. The assumptions for applying parametric statistical tests were satisfied for all variables.

### Behavioral Results

Results performed on behavioral ratings scores showed no significant group effect (*t* = -0.66, *p* > 0.5).

### EEG Results

**FIGURE 5:**
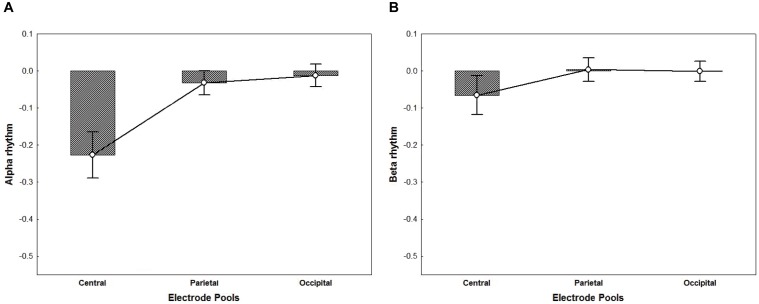
The plots represent the main effect of Electrode Pools for alpha rhythm (*p* < 0.001) **(A)** and beta rhythm (*p* < 0.05) **(B)** irrespective of groups. Negative values indicate suppression whereas a value of zero indicates no suppression and values greater than zero indicate enhancement. The ANOVA performed on alpha **(A)** and beta **(B)** activity showed that mu rhythm was significantly more suppressed in the Central Electrode Pool vs. the Parietal and the Occipital ones. Error bars represent standard error of mean (SE).

For the ANOVA performed on alpha activity (8–13 Hz), a significant main effect of Electrode Pool was found [*F*_(2,32)_ = 21.11, *p* < 0.001, η^2^_p_ = 0.57]. *Post hoc* comparisons showed that the alpha rhythm was significantly more suppressed in the Central Electrode Pool vs. the Parietal and the Occipital pools (*p*_s_ < 0.001) (Figure 5A). Likewise, for the ANOVA performed on beta activity (18–25 Hz) a significant main effect of Electrode Pool was found [*F*_(2,32)_ = 4.98, *p* < 0.05, η^2^_p_ = 0.24]. *Post hoc* comparisons showed that the beta rhythm was significantly more suppressed in the Central Electrode Pool vs. the Parietal and the Occipital ones (*p*_s_ < 0.05) (Figure 5B).

Alpha and beta suppression turned out to be area-specific – evident in the Central electrode pool only. Hence, group differences in the alpha and beta rhythm suppression for the Central electrode pool were investigated using two *t*-tests (two tailed, *p* < 0.05).

**FIGURE 6:**
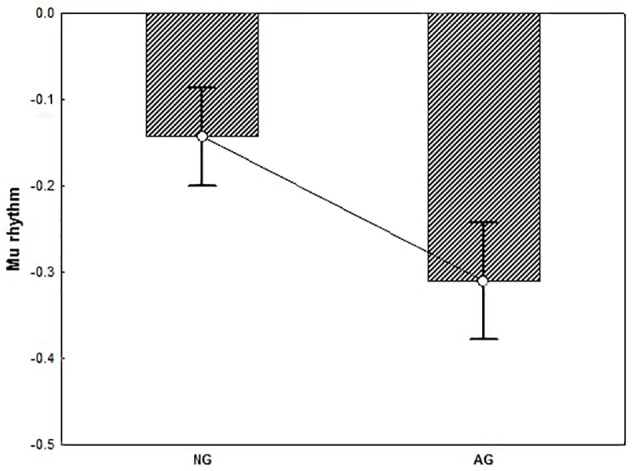
The plot represents the group difference (*p* < 0.05) for the *t*-test conducted on the alpha activity (mu rhythm) recorded from the Central electrode pool. Negative values indicate suppression. Result showed that mu rhythm was significantly more suppressed in AG vs. NG. Error bars represent standard error of mean (SE).

T-test performed on alpha activity revealed a significant group difference showing that alpha rhythm was significantly more suppressed in AG vs. NG (*t* = 2.16, *p* < 0.05) (Figure 6). T-test performed on beta activity on the other hand, yielded no significant results (*p* > 0.90).

### Behavioral Rating and Correlation Analysis Results

Independent samples *t*-test performed on behavioral ratings showed that AG were more familiar to the scenes presented in the experiment (*t* = 9.41, *p* < 0.001). Moreover, results revealed that AG perceived the scenes as more realistic with respect to NG (*t* = -2.46, *p* < 0.03). No group differences were found for behavioral ratings reporting the sense of being present in the scene and subjective amount of movement perceived (*p*_s_ > 0.80).

Separately for NG and AG, Spearman’s rho correlation analyses were performed between mu rhythm and behavioral ratings of Familiarity, Presence, and amount of Movement perceived in the scenes.

**FIGURE 7:**
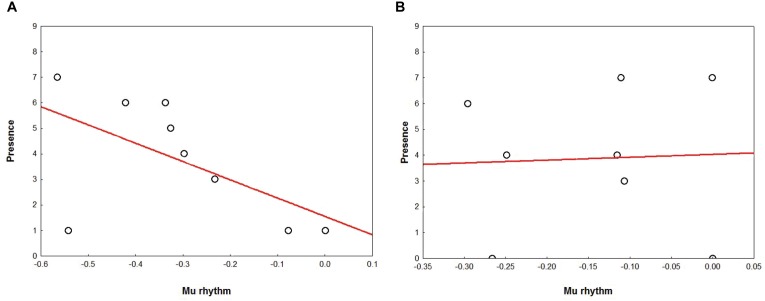
Correlation between the Presence ratings (Y axis) and mu suppression (X axis), in AG **(A)** and NG **(B)**. Plots show a tendency in the AG **(A)** for which the stronger the mu rhythm suppression, the higher the subjective rating of Presence.

In AG, results showed a marginally significant correlation (*r* = -0.64 *r^2^* = 0.41, *p* = 0.06) for which the stronger the mu rhythm suppression, the higher the subjective rating of Presence (Figure 7A). Correlation analyses performed for NG yielded no significant results (all *p*_s_ > 0.20) (Figure 7B).

## Discussion

In this study, we gauged the intensity of mu rhythm suppression from the cortical motor areas during the observation of artificial landing scenarios while performing a distance task in participants with flight expertise (pilots) and without flight expertise (novices).

Because of the overlap between mu and alpha activity, we controlled this matter by including not only experimental effects at the central electrode sites, but also the parietal and occipital sites (Perry and Bentin, 2009; Hobson and Bishop, 2016). In addition, given the multispectral nature of mu rhythm (Tiihonen et al., 1989; Hari, 2006), we investigated the reactivity of two relevant sensorimotor frequencies bands: alpha (8–13 Hz) and beta (18–25 Hz). We hypothesized that in pilots, previous experience with a landing scenario related with flight expertise would modulate scene perception by eliciting perception-action mechanisms at brain level.

First, in line with previous work (Babiloni et al., 2002) our preliminary results provided support for a topographic specificity of both mu and beta rhythms that, irrespective of groups, appeared to be located in the central electrode site vs. parietal and occipital sites.

A further analysis specifically targeting the central electrodes showed that group differences were evident for the alpha mu rhythm but not beta. Mu was found to be significantly more suppressed in pilots vs. novices, selectively in the alpha range activity. This is consistent with the claim that in pilots only, performing a distance task on a landing scenario evokes sensorimotor activation, as exemplified by mu rhythm suppression recorded from the central electrode site. Such a sensorimotor activation in pilots is supported by the results of the analysis performed on the parietal and occipital electrode sites, which suggests that mu rhythm suppression does not reflect posterior visual alpha activity.

To the best of our knowledge, this is the very first study that utilizes static stimuli to address possible brain signatures and specifically sensorimotor mechanisms related to flight expertise in pilots. Mirroring mechanisms indeed have been already reported in complex domains requiring flight experience in previous works that utilized dynamic scenarios (Callan et al., 2012, 2013). Notably, the pilots in our study reported that the stimuli were perceived as significantly more realistic compared to novices, notwithstanding the adoption of artificial scenes and the absence of any virtual reality aid. Complementing these findings, a tendency showed that the stronger the sensorimotor activation in pilots, the higher their perception of being present in the scene. This is in line with a previous study (Vecchiato et al., 2015) that reported how the sense of being in a virtual architectural environment was related to the involvement of sensorimotor mechanisms as measured by mu suppression. Notably, the sense of presence has been considered as an index of a physiological state which reproduces realistic behaviors and physiological responses as if the participant was experiencing a real-life situation (Vecchiato et al., 2015).

Why should a distance task in a landing scenario evoke such sensorimotor activity at brain level in pilots? It has been already observed how a distance task constitutes an implicit reachability task for a pilot (Langewiesche, 1944). This revitalized Gibson’s claim, in which “The psychology of aircraft landing (…) it is a *psychology of locomotion*, which occurs in time as well in space, and the problems are those of the judgments required for control of locomotion.” (Gibson et al., 1955). When a pilot is asked to gauge the distance of a given point on the ground in a landing scenario, he automatically perceives the scene in terms of *locomotion constraints*, which implies *action.* Supporting this idea, it has been demonstrated how reachability judgments for manipulable objects elicits a motor activity in the brain as measured by mu suppression (Wamain et al., 2016), wherein the involvement of the motor system in the perception of manipulable objects depends on the anticipation of interaction with them. In particular, reachability estimates trigger activation within a fronto-parietal network that overlaps with the one that is involved in the production of actual goal-directed movements (Bartolo et al., 2014; Wamain et al., 2016). These studies involved the perception of manipulable objects located in the peri-personal space and they demonstrated how they are automatically coded in motor terms at brain level (Cardellicchio et al., 2011; Coello et al., 2012; Iachini et al., 2014). It is also noteworthy to report that while the Mirror Neuron system has been to a large part associated with observation of action of the articulators like hand reaching and grasping (Caspers et al., 2010) the involvement of the Mirror Neuron system for action observation and execution has also been extended to include the use of complex tools (Arbib et al., 2009). Evidence supporting the extension of tool use to incorporate the Mirror Neuron system has been reported in some works (Ferrari et al., 2005; Jacobs et al., 2010). One of the novelties of our study is that sensorimotor mechanisms in pilots have been observed in static, artificial images, which display a landing scenario where the aiming point (i.e., the runway number) is represented well beyond the peri-personal space. Also, brain sensorimotor activation occurred in spite of lack of participants’ movement and irrespective of any subjective perceived movement in the scenarios. As said, a spatial dependency of action simulation has been reported for peri-personal space (Bartolo et al., 2014; Wamain et al., 2016). The peri-personal space is the area around the body where objects are coded in motor terms for the purpose of goal-directed actions. In pilots, we may expect that a possible extension of the reachable space (i.e., area of action) would occur, with the plane being a sort of tool allowing to reach – or *grasp* – the extra-personal space. The conceptualization of a landing task as a *locomotion* task (Gibson et al., 1955), possibly underpinned by brain sensorimotor mechanisms, aligns with the idea of the role of the body as a “vehicle for being-in-the-world” (Merleau-Ponty, 1962). Thus, in the context of flight expertise, can an airplane be then considered a sort of extension of a pilot’s body, in order to act upon the environment? Can the airplane be “embodied,” in order to perceptually grasp the world? Such a sensorimotor activation may be indeed considered as a form of embodied simulation (Gallese, 2003, 2005), defined as a functional mechanism characterized by the reuse of the one’s motor representation when observing any possible visual result of such actions. A possible explanation of our findings is that such embodiment mechanisms found in pilots might be evoked by the anticipation of interaction with the typical environment wherein pilots’ skillful performance normally takes place, with its corollary of perception-action chains required to achieve the goal of safe locomotion in a landing task (Cannon et al., 2014; see Jola et al., 2012 for an alternative hypothesis).

Several limitations of the study should be taken into account when considering final results. First, the modest sample size might have affected the statistical power and the possibility to extend the results to the general population. Second, the electromyographic activity of participants was not controlled. However, participants were asked to remain seated without moving their arms, legs, and hands during stimuli exposure. During data collection, the experimenter monitored participants’ behavior and those who exhibited movement and muscular activity in the EEG were discarded. Hence, the activity detected over the motor areas could have been determined only by processes that are not related with actual movement execution.

An alternate interpretation of our results is that mu suppression may occur after the percept is formed, and thus in this case, it would merely reflect motor preparation with no active role in the perceptual process. In this respect, it is important to consider that we avoided utilizing any sort of motion-related aspect in our study, by asking participants to perform a simple distance estimation task during static (vs. dynamic) stimuli perception. This was done on purpose in order to avoid any motion-related effect on brain activity. Moreover, the lack of any group effect in beta power supports the idea that the stronger mu suppression found in pilots was not related to motor preparation or motor imagery (McFarland et al., 2000). Another aspect worth reporting is that pilots and novices didn’t differ when asked to provide an explicit judgment of the amount of movement perceived in the scenes. However, further studies are warranted in order to better clarify this aspect. In particular, Event Related Potential (ERP) studies that utilize anticipatory potential markers can be useful to this end, as ERPs reliably precede movement execution and can hence index motor activation electrophysiologically (Nam et al., 2011).

Another potential confound in our study resides on potential mixing of activity of brain sources across all electrodes, for which we cannot completely rule out some contribution of alpha activity distinct from mu rhythm coming from other brain regions. To overcome this issue, analysis over source localized activity in motor, occipital and parietal areas is necessary in future studies to make stronger conclusions.

Additionally, the participants in our study were all males except from one female in the non-expert group. This likely happened because of the prevalence of males in pilot population, and this issue can be a potential confound in our study. However, a prior study did not provide support for gender effects in mu suppression on activity at central sites during action observation (Hobson and Bishop, 2017).

Moreover, studying lateralization effects was beyond the aim of this study. However, we put efforts in order to balance handedness between the two groups. It is worth to report that previous studies did not find any laterality effects for alpha and beta rhythms during movement (Babiloni et al., 2002) and work of art (Umilta’ et al., 2012) observation.

In sum, these findings provide empirical support for the role of embodied simulation mechanisms in visual perception in the aviation context, and add important insights on how such brain mechanisms may be related to flight expertise (Kasarskis et al., 2001; Taylor et al., 2007; Charness and Tuffiash, 2008). This is in line with a neuroergonomic approach, which brings together contributions from ecological psychology and embodied cognition. Such an approach to answering this research question in the aviation context presents many advantages. First, it emphasizes the situated nature of perceptual experience, which makes the aspects of embodiment and relational embeddedness in the world essential to fully understand how people engage with environments. This view suggests that the way in which we interact with the environment depends on action capabilities and the potential for action (Gibson, 1969). It follows that an embodied cognition approach has the capacity to account for situations even when the “body” includes tools or technologies such as an aircraft. Moreover, one of the main advantages of a neuroergonomic approach resides in the use of implicit measures – referred as “neuro-markers.” These neuro-markers may provide a potential index for flight expertise. A first key application of this approach is to utilize such neuro-markers in order to guide training and to enable expert performance (Sestito et al., 2018). These neuro-markers furthermore, can potentially quantify in an objective way the interaction between pilot and aircraft, and may ultimately be used to optimize this interaction. This notably, will add toward improving the way humans interact with or control complex dynamic systems (Parasuraman and Wilson, 2008), where deviations from expected behaviors can lead to human error. Indeed, there is an emerging interest in applying neuroscientific findings to increase the effectiveness of the interaction between the human operators and *ad-hoc* designed devices (i.e., human-machine interfaces, HMI) to improve flight safety (see Aricò et al., 2017a for a review, and Aricò et al., 2017b for possible applications in brain-computer interfaces). The actionable implication of Gibson’s concept of affordances indeed, lies in the entailed property of constraints on motor units for a given task, and the possibility to utilize Mirron Neuron activity as a neurophysiologic index of motor expertise that is required in order to successfully perform a given task. Previous work illustrated how this property can be easily implemented in ecologically-inspired HMI design in the flight deck (Roesler et al., 2016) where visual properties will directly specify affordances to guide behavior in a given context, hence supporting skilled performance and fast decision-making processes (Wiggins and O’hare, 1995; Sestito et al., 2018). Overall, such perspectives on possible applications of the neuroergonomic approach in a real-world aviation realm appear promising and warrant further investigations into neuroergonomics.

## Author Contributions

MS conceived and designed the study, wrote the protocol, set up the experiments, collected, analyzed, and interpreted the data, and drafted the manuscript. AH designed the study, set up the experiments, interpreted the data. JN set up the experiments and analyzed the data. JF conceived and designed the study, and interpreted the data. All the authors contributed to the final revision of the manuscript.

## Conflict of Interest Statement

The authors declare that the research was conducted in the absence of any commercial or financial relationships that could be construed as a potential conflict of interest.

## Footnotes

1 http://www.unity3d.com

2 http://www.neurobs.com

3 http://www.brainproducts.com

4 http://www.brainproducts.com
