# Supplementary material for: Investigating Neural Sensorimotor Mechanisms Underlying Flight Expertise in Pilots: Preliminary Data From an EEG Study
Source: Front Hum Neurosci. 2018 Dec 13;12:489. doi: 10.3389/fnhum.2018.00489 (PMC6300503; doi:10.3389/fnhum.2018.00489)
Supplement: Supplementary file 1 [file Data_Sheet_1.docx]

**STIMULI VALIDATION STUDY**

The following study was intended to validate the height/distance ratios adopted in the main study. Stimuli construction for this aspect was inspired by the FAA Airplane Flying Handbook (see Figure 2 in the main text, upper panel), according to the idea of showing different glide paths – a low, a correct, and a high path.

The accuracy with respect to distance estimation, on the other hand, was essentially of no interest in this study for two reasons. First, the nature of the stimuli itself (i.e. static scenarios artificially created, with no clues present in the scene to aid in distance estimation) cannot really provide a realistic, ecological scene for this purpose. Second and relatedly, the main scope of the study was to investigate the brain activity while performing a given task – a distance estimation task – irrespective of actual accuracy.

**MATERIAL AND METHODS**

**Participants**

Thirteen pilots (mean age 46.50, DS ± 17.70; mean education 14.00, DS ± 3.34; 11 males) were asked to respond to a Qualtrics^TM^ Survey aimed at validating the stimuli intended to be subsequently utilized in the main electrophysiological study. These participants were active members of a local flight school (Top Gun Fly School) located in Reggio Emilia Italy, and included recreational and professional pilots with variegated levels of expertise.

General information was acquired from participants and included gender, age, handedness, and education. As a measure of pilot’s expertise, the number of flight hours (VFR and IFR) and years of flight experience were recorded. Demographic characteristics of the sample are reported in the Supplementary Table 1.

**Table 1.** Demographic features of the pilots involved in the validation study.

| **Characteristic** |  | **Mean/ Ratio** |  | **SD** |  | **Range** |
| --- | --- | --- | --- | --- | --- | --- |
|  |  |  |  |  |  |  |
| **Age (years)** |  | 46.50 |  | 17.70 |  | 18-70 |
| **Education (years)** |  | 14.00 |  | 3.34 |  | 8-22 |
| **Males:females** |  | 11:2 |  |  |  |  |
| **Right:left handedness** |  | 12:1 |  |  |  |  |
|  |  |  |  |  |  |  |
| **Flight expertise** |  |  |  |  |  |  |
| VFR^a^ (hours) |  | 826.31 |  | 1,341.13 |  | 23-4,000 |
| IFR^b^ (hours) |  | 769,23 |  | 2,773.50 |  | 0-10,000 |
| Total flight hours |  | 1,595.54 |  | 3,844.53 |  | 23-14,000 |
| Flight experience (years) |  | 13.08 |  | 16.63 |  | 0-50 |

^a^ VFR: Visual Flight Rules

^b^IFR: Instrument Flight Rules

**Stimuli**

Twelve static images depicting a typical landing scenario were constructed using the Unity game engine (63° field of view) (<http://www.unity3d.com>). Stimuli represented a runway being seen from three different increasing distances (30 m/98 feet, 90 m/295 ft, 150 m/689 ft) and three height/distance ratios corresponding to three different glide paths (0.2 = low glide path, 0.3 = on the glide path, 0.4 = high glide path; see Figure 2 in the main text for stimuli example). Based on this, in a typical low glide path view (i.e. 0.2 height/distance ratio), the approach is too low and the runway cannot be reached in a powerless glide. While a typical correct landing approach is represented in a scenario adopting the 0.3 height/distance ratio (i.e. on the glide path), the other possible scenario is represented by a high approach (i.e. 0.4 height/distance ratio), where the runway is reachable though the landing spot is shifted forward.

**Procedure**

Participants were invited to respond to a Qualtrics^TM^ Survey. Twelve stimuli were randomly presented, repeated three times each. For every stimulus, participants were asked to:

1. Estimate the distance to the runway in meters;
2. Estimate the reachability of the runway in a powerless glide;
3. Express how familiar they are with the scenes presented;
4. Indicate how realistic they perceive the scenes to be;
5. Express to what extent did they have the sense of being in the scene - that is, to what extent are there times during the experience when the scene becomes reality for them and they almost forget about the real world in which the whole experience was really taking place (see Vecchiato et al., 2015);

Questions 2-5 were answered by utilizing a 0-9 Likert scale.

**Rating data analysis**

The rating scores of each participant were averaged on the basis of distance and height/distance ratios. The corresponding averaged rating scores were entered into a 3 (Distance: 30 m, 90 m, 150 m) × 3 (Height/distance ratios: 0.2, 0.3, 0.4) repeated measures ANOVA (p < 0.05), with Distance and Height/distance ratios as within-participants factors.

For performed analyses, p values < 0.05 were considered to be statistically significant. Post-hoc comparisons (Bonferroni corrected for multiple comparisons) were applied on significant main effects and interactions.

**RESULTS**

First, normal distribution of variables was evaluated through visual inspection of histograms and the application of the Kolmogorov-Smirnov test. It turned out that the assumptions for applying parametric statistical tests were met for all variables.

Results obtained from Distance estimation showed a main effect of Distance (*F*_(2,22)_ = 74.28, *p* < 0.001). *Post hoc* comparisons confirmed that the three distances adopted (i.e. 30 m, 90 m, 150 m) were correctly perceived as increasing distances by all participants, irrespective of height/distance ratios (all p_s <_ 0.001). Moreover, the Height/Distance Ratio factor was found to be significant (*F*_(2,22)_ = 6.69, *p* < 0.001). *Post hoc* comparisons showed that the height/distance ratio 0.4 was perceived as being closer than height/distance ratio 0.2 and 0.3. Finally, the Distance x Height/Distance Ratio interaction was significant (*F*_(2,22)_ = 4.48, *p* < 0.005). *Post hoc* comparisons showed that 30 m distance was perceived as closer with respect to distance 90 m and 150 m for all Height/Distance Ratios (all p_s <_ 0.001). Whereas, distance 90 vs. 150 m was perceived closer for 0.2 height/distance ratio only (all p_s <_ 0.005).

Overall, results on Distance ratings showed that participants committed an overestimation with respect to the effective parameters that have been set for stimuli capture. However, as mentioned above, accuracy in distance estimation was not considered essential for the aim of the present study.

Most importantly, results obtained for reachability judgments showed a main effect of Height/Distance Ratio (*F*_(2,18)_ = 17.89, *p* < 0.001). As expected, p*ost hoc* comparisons showed that the height/distance ratio 0.2 (i.e. low glide angle) was perceived as being significantly less reachable vs. height/distance ratios 0.3 and 0.4 (*p* < 0.001) (Figure 1). The interaction Distance x Height/Distance Ratio was also significant (*F*_(4,36)_ = 2.70, *p* < 0.05). However, p*ost hoc* comparisons showed no significant effects.


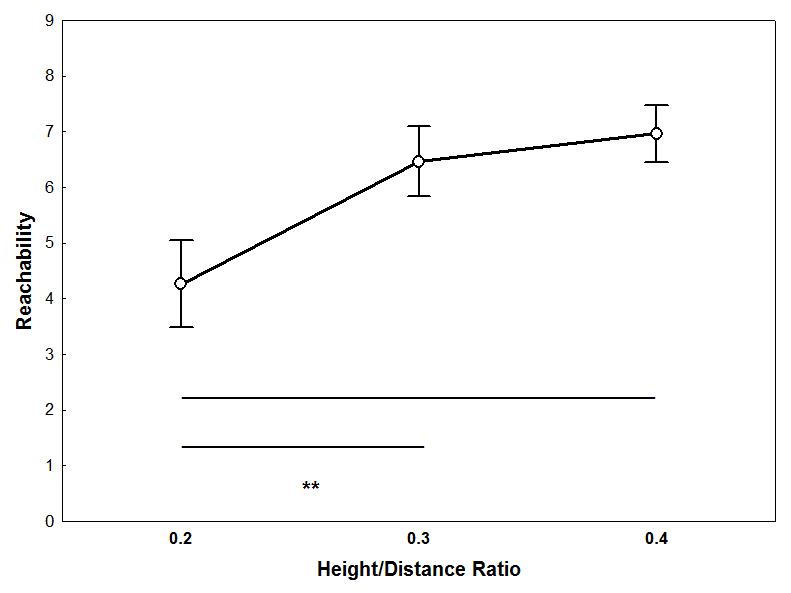


**Figure 1.** The plot represents the main effect of Height/Distance Ratio (*p* < 0.001). The height/distance ratio 0.2 (i.e. low glide angle) was perceived as being significantly less reachable vs. height/distance ratios 0.3 and 0.4 (*p* < 0.001). Error bars represent standard error of mean (SE).

Finally, results obtained from Familiarity ratings, Presence ratings, and the degree of how realistic the scenes were perceived, showed no significant main effects and interactions (all p_s >_ 0.20). This confirmed that ratings were homogeneous for all distance and height/distance ratio conditions.
